# Exploring Disparities in Healthcare Wearable Use among Cardiovascular Patients: Findings from a National Survey

**DOI:** 10.31083/j.rcm2411307

**Published:** 2023-11-09

**Authors:** Ranganathan Chandrasekaran, Pratik Sharma, Evangelos Moustakas

**Affiliations:** ^1^Department of Information & Decision Sciences, University of Illinois at Chicago, IL 60607, USA; ^2^Edinburgh Business School, Heriot-Watt University, Dubai 38103, UAE

**Keywords:** wearable devices, cardiovascular care, socio-economic factors, health factors, digital literacy, wearable data sharing

## Abstract

**Background:**

Use of healthcare wearable devices holds significant potential for improving the prevention and management of cardiovascular diseases (CVD). However, we have limited knowledge on the actual use of wearable devices by CVD patients and the key factors associated with their use. This study aims to assess wearable device use and willingness to share health data among CVD patients, while identifying socio-demographic, health, and technology-related factors associated with wearable technology use.

**Methods:**

Using a national survey of 933 CVD patients, we assess use of wearable healthcare devices (use, frequency of use and willingness to share health data from wearable with a provider), and a set of socio-demographic factors (age, gender, race, education and household income), health-related variables (general health, presence of comorbid conditions: diabetes and high blood pressure, attitude towards exercise) and technology self-efficacy using logistic regression.

**Results:**

Of the 933 CVD patients, 18.34% reported using a healthcare wearable device in the prior 12 months. Of those, 41.92% indicated using it every day and another 19.76% indicated using it ‘almost every day’. 83.54% of wearable users indicated their willingness to share health data with their healthcare providers. Female CVD patients are more likely to use wearables compared to men (odds ratio (OR) = 1.65, 95% confidence interval (CI) = 1.04–2.63). The odds decrease with age, and are significantly high in patients with higher income levels. In comparison with non-Hispanic White, Hispanic (OR = 0.14, 95% CI = 0.03–0.70) and African Americans (OR = 0.17, 95% CI = 0.04–0.86) are less likely to use healthcare wearables. CVD patients who perceive their general health to be better (OR = 1.45, 95% CI = 1.11–1.89) and those who enjoy exercising (OR = 1.76, 95% CI = 1.22–2.55) are more likely to use wearables. CVD patients who use the internet for searching for medical information (OR = 2.10, 95% CI = 1.17–3.77) and those who use electronic means to make appointments with their providers (OR = 2.35, 95% CI = 1.48–3.74) are more inclined to use wearables.

**Conclusions:**

Addressing low wearable device usage among CVD patients requires targeted policy interventions to ensure equitable access. Variations in gender, age, race/ethnicity, and income levels emphasize the need for tailored strategies. Technological self-efficacy, positive health perceptions, and exercise enjoyment play significant roles in promoting wearable use. These insights should guide healthcare leaders in designing effective strategies for integrating wearables into cardiovascular care.

## 1. Introduction

Cardiovascular diseases (CVD) are a group of disorders that affect the heart and blood vessels. It includes conditions such as coronary heart disease, cerebrovascular disease, and peripheral arterial disease. CVD is a leading cause of mortality globally, especially in developed countries like the United States. According to the US Center for Disease Control and Prevention [1], about 697,000 people in the United States died from heart disease in the year 2020, which is 1 in every 5 deaths. The financial burden of heart disease in the United States was about $229 billion, which includes the cost of healthcare services, medicines, and lost productivity due to death [2].

Wearable healthcare devices such as fitness trackers, smartwatches, and electrocardiogram patches have enormous potential for improving cardiovascular health outcomes by offering novel digital health services and interventions [3]. These devices can monitor physiological changes such as heart rate, physical activity, and sleep quality in real-time [4]. Wearable sensors can help people with atrial fibrillation monitor their pulse and heart rate [5]. Wearable-generated heart rate measurements can potentially be incorporated into cardiovascular risk scores. Wearable devices can provide a constant stream of healthcare data for disease diagnosis and treatment by actively recording physiological parameters and tracking metabolic status.

In recent years, wearable smartwatches have exhibited their potential in prevention, assessment and continuous patient monitoring, aiding in diagnosis and treatment of CVD problems, and offering various assistance functionalities [6, 7, 8]. Recognizing their potential, several technology companies such as Apple (Apple Watch), Samsung (Galaxy), and Google (Fitbit) have incorporated smartwatch features that can monitor and transfer several biometrics parameters that can aid cardiologists and physicians. These devices can monitor several physiological parameters, including blood pressure, oxygen saturation, heart rate, pulse, sleep patterns, physical activity, and more [9]. The incorporation of photoplethysmographic (PPG) sensors in smartwatches has led to a significant increase in community-based heart rate monitoring. With the latest smartwatch models, the PPG sensor can detect not only pulse rate but also irregularities in heart rhythm. Furthermore, smartwatches can serve as reminders for daily activities such as exercise regimens and medication schedules. Additionally, they can track physical movements, such as a daily step count and global positioning system (GPS) location. Studies have documented the diagnostic accuracy of smartwatch wearables in detecting cardiac arrhythmias [5, 10, 11] and ventricular tachycardia [12] and a growing number of studies have argued for potential future uses of smartwatches in cardiac care [13, 14]. Use of wearable devices and trackers can greatly improve participation in physical activity and reduce sedentary behavior in CVD patients [15]. Furthermore, acceptance and utilization of healthcare wearables by CVD patients are essential for laying the groundwork for advanced healthcare applications in the future, incorporating technologies like the metaverse and blockchain in CVD care [16, 17].

Effective adoption and utilization of wearable healthcare devices by patients, along with their willingness to share the generated data with healthcare providers, are crucial factors for harnessing the full potential benefits of these devices. Studies have reported that roughly one-third of USA individuals adopting a wearable medical device [18, 19], and the majority of individuals who owned a device were willing to share data with their providers. Patients with certain conditions, such as diabetes and hypertension, were more likely to adopt devices and share data with providers. Social determinants of health, such as income and usual source of care, negatively affected wearable device adoption and data sharing. Patient uptake of these devices could be affected by barriers such as safety issues, confidence, functionality, affordability and other risks [20].

**Fig. 1.**
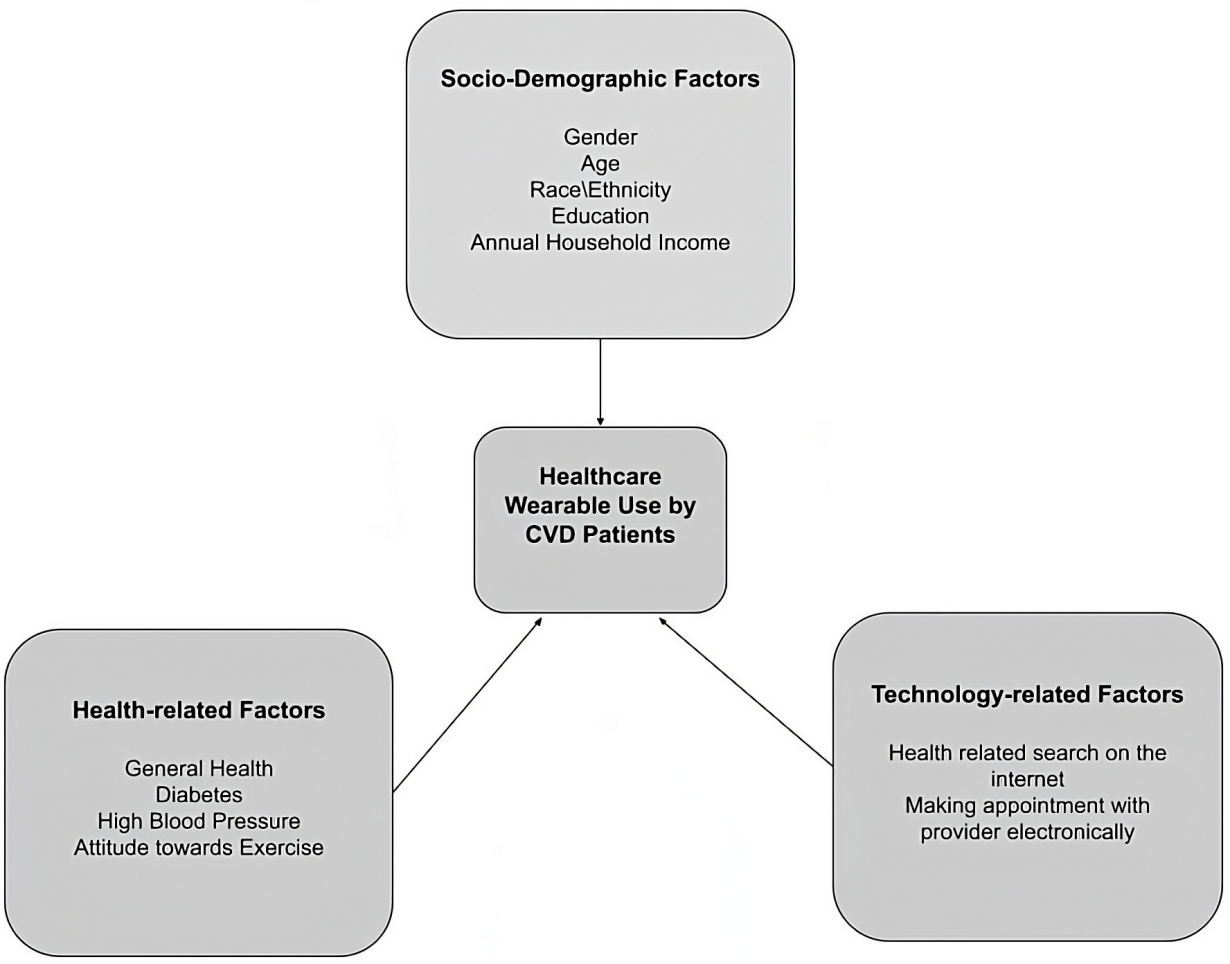
**Key factors associated with healthcare wearable use among cardiovascular diseases (CVD) patients**.

To harness the potential benefits of wearable healthcare devices for cardiovascular care, an in-depth understanding of the patterns in use and the key predictors of wearable device usage among CVD patients is needed. Though there are survey-based studies that have examined patients’ intentions to use wearables [21, 22, 23, 24], studies using national-level data on wearable use among specific groups such as CVD patients are sparse [21, 22, 23, 24, 25]. Addressing this gap, this study examines the use of wearable healthcare devices among CVD patients using cross-sectional survey data collected by the National Cancer Institute’s Health Information National Trends Survey (HINTS)-5 Cycles 3 and 4. Our research objectives (summarized in Fig. 1) are (i) to examine the extent of wearable device adoption by CVD patients, and their willingness to share health data collected from wearables with providers, and (ii) to examine the key socio-demographic and health related factors that are associated with wearable technology use by CVD patients.

## 2. Materials and Methods

### 2.1 Data

This study utilized data from the National Cancer Institute’s Health Information National Trends Survey (HINTS) - Cycles 3 and 4, conducted in 2019 and 2020 respectively. The survey collected information through self-administered postal questionnaires and a web-pilot. The data from both cycles were merged using the provided tool on the HINTS website [26]. HINTS is a nationally representative survey targeting U.S. adults aged 18 years or older in non-institutionalized settings, aiming to gather comprehensive data on health-related information needs, healthcare access, usage patterns, behaviors, perceptions, and knowledge related to health. To ensure inclusivity, the survey employed a stratified sampling method, considering areas with high and low concentrations of minorities. Participants received questionnaires by mail, with reminders and follow-ups for non-respondents. Additional information on the survey methodology and comprehensive data collection reports can be found on the HINTS website (https://hints.cancer.gov/data/methodology-reports.aspx).

The HINTS survey utilized a 2-stage, stratified, probability sampling approach. Residential addresses in the US Marketing Systems Group database were categorized based on the density of racial and ethnic minority populations, resulting in high and low minority population areas. A random subset of addresses was selected, with an oversampling of high-level minority addresses to improve representation. One adult from each selected household participated, and comprehensive weights were calculated to address nonresponse and noncoverage. These weights were adjusted to align with population totals from the U.S. Census Bureau’s American Community Survey (ACS), considering factors like age, gender, education, race, ethnicity, and census region [27]. Replication weights were computed using the jackknife approach to ensure reliable statistical outcomes. The HINTS survey has been a valuable resource for researchers investigating various health behavior topics, including the use of wearable devices [18, 27, 28].

For data analysis, we included all CVD patients who answered “yes” to the question ‘Has a doctor or a health professional ever told you that you had a heart condition such as heart attack, angina or congestive heart failure?’. This resulted in a final dataset of 933 respondents with CVD conditions.

### 2.2 Variables

The study focused on two main variables of interest: (i) Use or non-use of a healthcare wearable device in the previous twelve months, and (ii) Willingness to share data generated by the wearable device with healthcare providers. Both variables were measured as binary responses (yes/no) through specific questions that assessed whether respondents had used any wearable device (such as Fitbit, Apple Watch, or Garmin Vivofit) to monitor or track their health or activity in the previous twelve months. Similarly, users were also asked to indicate their willingness to share health data collected by their wearable device with a healthcare provider.

Past research studies have identified a variety of demographic and socioeconomic factors that could be associated with an individual’s adoption and use of health technologies. Gender, race and ethnicity, income, and education levels have been found to affect adoption of eHealth [29], personal health records [30], telemedicine [31], and wearable devices [18]. In line with this cohort of studies, we include the following demographic variables for CVD patients in our dataset: gender, age, race/ethnicity, education, and annual household income.

We used three variables to capture the health conditions of CVD patients: (i) self-reported general health status that was measured using a single item asking individuals to rate their health as “1-poor”, “2-fair”, “3-good, “4-very good fair” or “5-excellent”, (ii) comorbid conditions of diabetes and (iii) blood pressure. These were coded as 1 or zero depending on the response provided to the question asking if there was diagnosis and subsequent communication from a health professional about diabetes and high blood pressure.

Technological self-efficacy was measured through a two-item assessment that focused on the utilization of technology by CVD patients for health-related purposes. These items inquired whether participants had employed a computer, smartphone, or other electronic means within the preceding 12 months to (i) search for health or medical information for themselves (yes/no), and (ii) use the internet for scheduling appointments with a healthcare provider (yes/no). Attitudes towards exercise were evaluated using a Likert-scale item which asked respondents to indicate their level of enjoyment in exercising, ranging from ‘1-not at all’ to ‘2-a little’, ‘3-some’, and ‘4-a lot’.

### 2.3 Data Analysis

Survey sampling is a method of selecting a subset of individuals from a larger population to gather information about the population. Drawing population-level inferences from a sample involves using statistical methods to estimate characteristics of the population based on the information gathered from the sample. This requires careful consideration of the sampling method used, as well as the potential for bias and variability in the sample. To make statistically valid inferences about the population, it is crucial to account for the sample design process during statistical analyses. While studies that rely on random sampling can utilize conventional statistical techniques, these techniques become invalid when applied to data from other sample designs. For instance, complex survey designs involving stratification, clustering, and unequal weighting require specialized techniques to produce appropriate estimates and standard errors [32].

In the case of the HINTS survey, probabilistic sampling was used to elicit responses from participants. To account for this sample design and calculate nationally representative estimates, STATA survey procedures incorporating the jackknife variance estimation technique and HINTS-supplied survey weights were applied. The survey design was declared, and weights were applied before performing the analyses. Logistic regressions were then used to examine the associations between the predictors of interest and the wearables use. STATA 16.1 (StataCorp LLC, College Station, TX, USA) software was used for performing the statistical analyses.

## 3. Results

Of the 933 CVD patients, 168 (18.34%) individuals reported using a healthcare wearable device in the prior 12 months, and the other 748 (81.66%) were non-users. Though the popularity and sales of smartwatches and activity trackers have experienced significant growth in the USA, our findings indicate that only a small proportion of CVD patients use wearable healthcare devices. Among the CVD patients who use wearable devices, 41.92% indicated using it every day, 19.76% indicated using it ‘almost every day’, 7.78% reported using it 1–2 times a week, 8.98% used it less than once a week, and 21.56% not using it at all. Moreover, 83.54% of wearable users indicated their willingness to share health data with their healthcare providers.

**Fig. 2.**
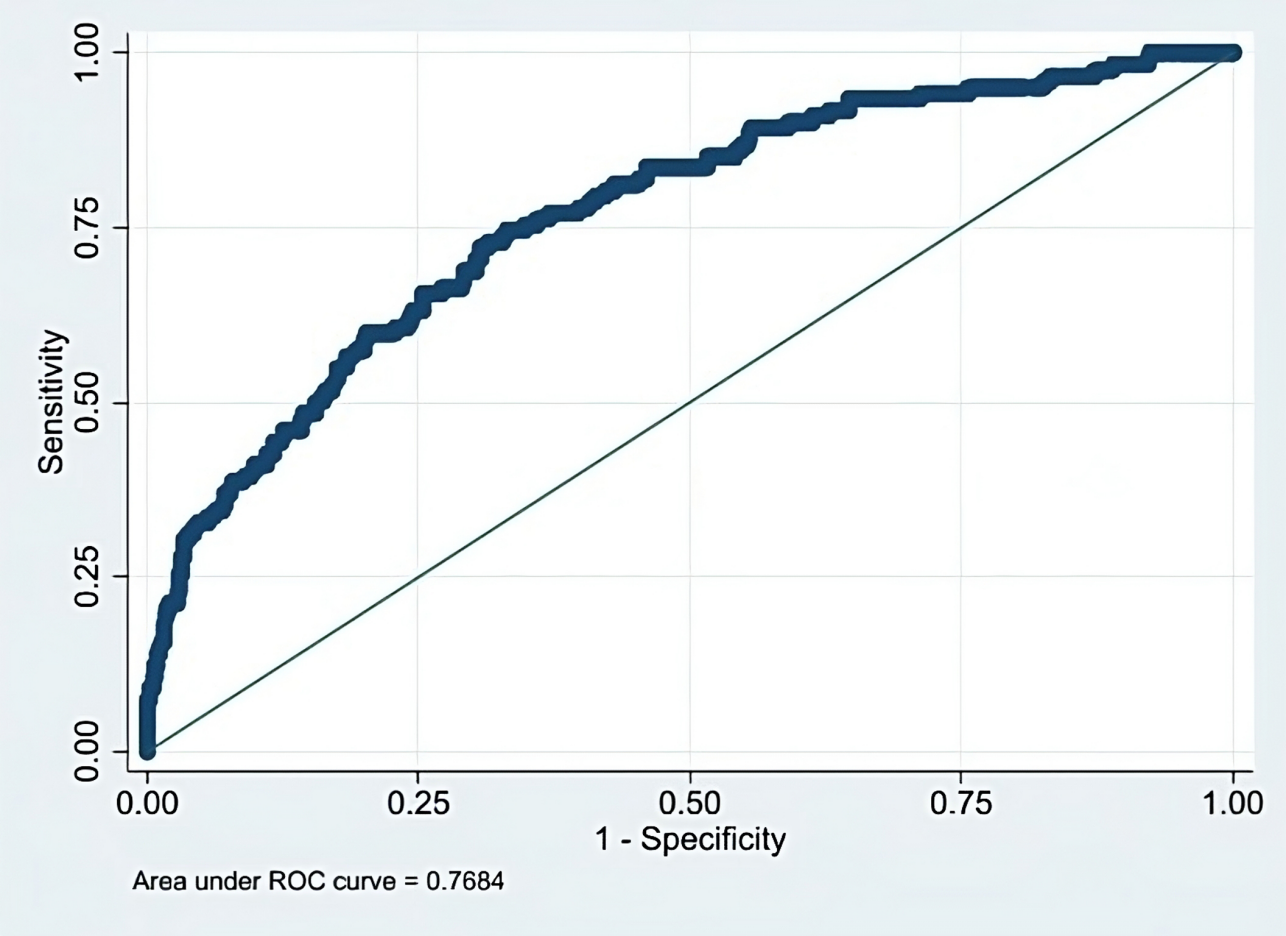
**Receiver operating characteristic (ROC) curve for logistic regression analysis**.

The socio-demographic characteristics of CVD patients in the dataset is presented in Table 1. The profile of CVD patients who used wearable healthcare devices in the past twelve months is also shown in Table 1. To further investigate the key factors associated with healthcare wearables use among CVD patients, we utilized logistic regression as our analytical method of choice. This was appropriate as our primary variable of interest was binary (indicating wearables use or non-use), while our predictor variables encompassed a combination of categorical and continuous variables. To diagnose collinearity amongst the variables, we examined variance inflation factor (VIF) values computed by the collin package in STATA. The VIFs for predictor variables ranged between 1.07 and 1.58, with an average VIF of 1.21, indicating that collinearity was not a problem. The logistic regression results, illustrating the associations between these factors and wearables use, are summarized in Table 2. The model fit, as assessed by pseudo-R square values from Efron’s $R^{2}$ is 0.17, Tjur’s D is 0.16, and Chi-squared = 528.603, *p* < 0.001. The receiver operating characteristic (ROC) curve associated with the results of logistic regression is shown in Fig. 2.

**Table 1.** **Profile of the sample, US population estimates and wearable device use**.

Respondent characteristics		Sample	US population ${\mathrm{estimates}}^{1}$	Use of wearable healthcare device in past 12 months in sample	
				Yes %	No %
Gender					
	Male	446 (53.48%)	10,640,024 (58.4%)	76 (49.35%)	363 (54.34%)
	Female	388 (46.52%)	7,577,117 (41.6%)	78 (50.65%)	305 (45.66%)
Age					
	18–34	24 (2.64%)	2,214,554 (11.2%)	12 (7.59%)	12 (1.63%)
	35–49	45 (4.96%)	1,596,190 (8.1%)	16 (10.13%)	29 (3.95%)
	50–64	224 (24.67%)	6,730,771 (34.2%)	40 (25.32%)	181 (14.63%)
	65–74	297 (32.71%)	4,370,828 (22.2%)	46 (29.11%)	247 (33.61%)
	75+	318 (35.02%)	4,784,854 (24.3%)	44 (27.85%)	266 (36.19%)
Education					
	Less than high school	106 (11.76%)	2,425,092 (12.2%)	7 (4.40%)	97 (13.34%)
	High school	216 (23.97%)	5,185,062 (26.2%)	29 (18.24%)	181 (24.90%)
	Some college	309 (34.3%)	9,142,969 (46.2%)	57 (35.85%)	248 (34.11%)
	College graduate	270 (29.97%)	3,048,394 (15.4%)	66 (41.51%)	201 (27.65%)
Race/Ethnicity					
	Non-Hispanic White	530 (67.52%)	11,980,283 (70.1%)	100 (69.44%)	423 (67.04%)
	Non-Hispanic Black	103 (13.12%)	1,716,086 (10.0%)	21 (14.58%)	81 (12.84%)
	Hispanic	104 (13.25%)	2,796,643 (16.4%)	15 (10.42%)	89 (14.10%)
	Non-Hispanic Asian or others	48 (6.11%)	602,752 (3.5%)	8 (5.56%)	38 (6.02%)
Annual household income					
	Less than $20k	229 (27.96%)	4,616,643 (25.5%)	25 (16.67%)	201 (30.59%)
	$20k to <$35k	142 (17.34%)	3,109,482 (17.2%)	17 (11.33%)	122 (18.57%)
	$35k to <$50k	127 (15.51%)	4,386,536 (24.2%)	22 (14.67%)	101 (15.37%)
	$50k to <$75k	138 (16.85%)	2,564,523 (14.2%)	32 (21.33%)	106 (16.13%)
	$75k or more	183 (22.34%)	3,444,045 (19.0%)	54 (36.0%)	127 (19.33%)

^1^Based on the estimates in 2019 and 2020 from the American Community Survey by the US Census Bureau. See HINTS website. US, the United States; HINTS, the Health Information National Trends Survey.

**Table 2.** **Logistic regression results: key socio-economic demographics, health and technology-related factors associated with wearable use by CVD patients**.

Factors		Use of healthcare wearable device in the last twelve months		
		Odds ${\mathrm{Ratio}}^{1\,a}$	[95% CI]	
Gender	Ref: Male			
	Female	1.65**	1.04	2.63
Age				
	Ref: 18–34			
	35–49	0.65	0.19	2.15
	50–64	0.32**	0.11	0.96
	65–74	0.25***	0.09	0.73
	75+	0.24***	0.08	0.72
Education	Ref: Less than high school			
	High School Graduate	1.31	0.45	3.85
	Some College	1.37	0.49	3.82
	College Graduate or more	1.26	0.44	3.63
Race/Ethnicity	Ref: Non-Hispanic White			
	Non-Hispanic African American	0.17***	0.04	0.86
	Hispanic	0.14***	0.03	0.70
	Non-Hispanic Asian or Other	0.31	0.06	1.59
Household Income	Ref: <20k			
	$20k to <$35k	1.05	0.46	2.45
	$35k to <$50k	1.47	0.66	3.26
	$50k to <$75k	2.04**	0.93	4.49
	>$75k	2.99***	1.41	6.33
General health status		1.45***	1.11	1.89
Diabetes		1.24	0.77	2.00
High Blood Pressure		0.64	0.35	1.08
Attitude towards Exercise		1.76***	1.22	2.55
Electronic Self Health Information Seeking		2.10***	1.17	3.77
Making appointments with provider electronically		2.35***	1.48	3.74

** *p* < 0.05; *** *p* < 0.01. ^a^ Odds ratios and 95% confidence intervals (CI) generated from multivariate logistic regression. Bold indicates odds ratios that are significant at *** <0.001, ** <0.01, and * <0.05. CVD, cardiovascular diseases.

**Fig. 3.**
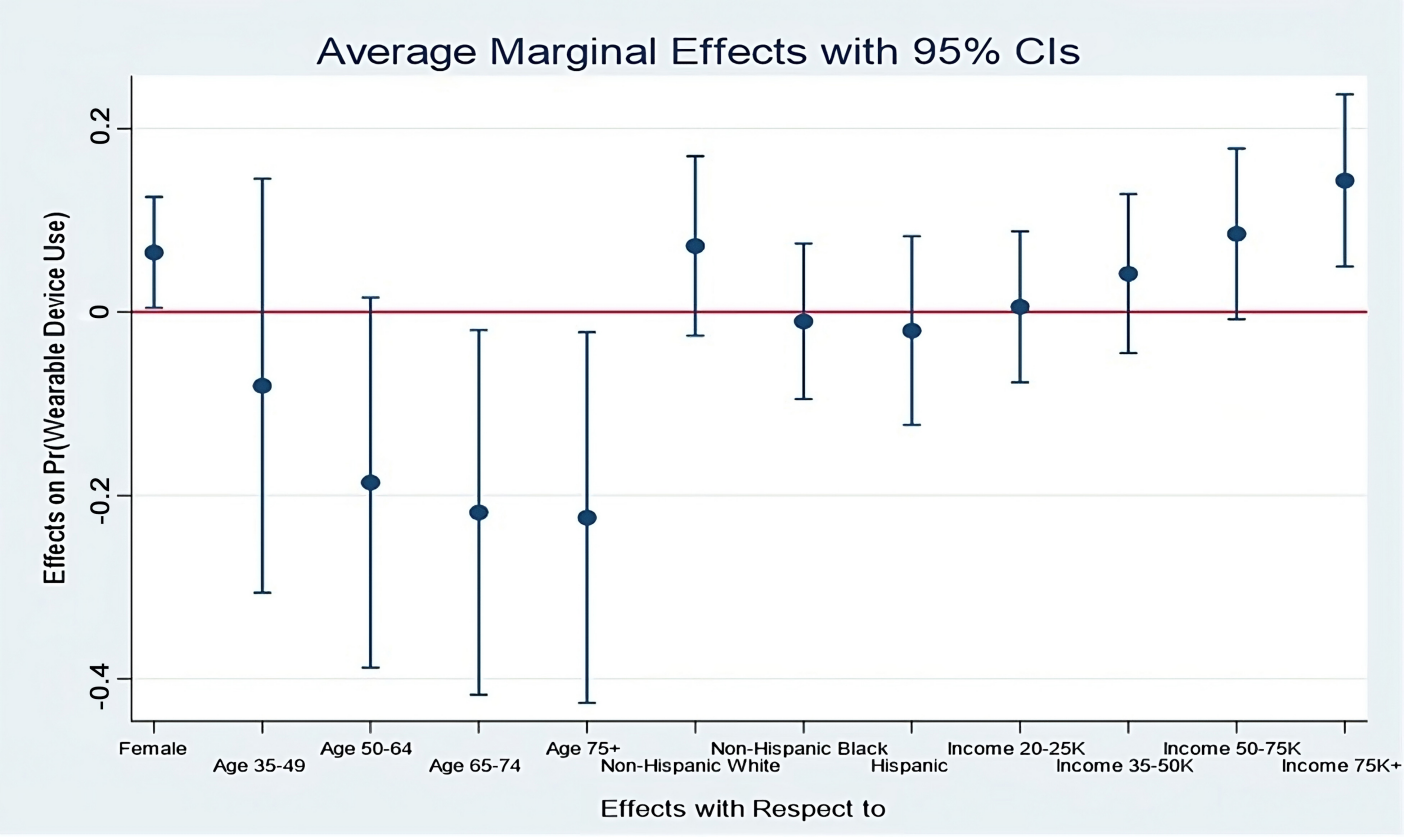
**Average marginal effects of key demographic variables on wearables use by CVD patients**. CI, confidence intervals; CVD, cardiovascular diseases.

Our logistic regression analysis (Table 2) revealed that gender, age, race/ethnicity, and annual household income were significant predictors of wearable use among CVD patients. The area under the ROC curve was 0.7684 (Fig. 2), indicating a good predictive ability of the model. Additionally, we analyzed the average marginal effects of the significant socioeconomic-demographic variables on wearable use, and these findings are presented in Fig. 3.

Of the demographic variables, logistic regression results indicated that female CVD patients are more likely to use healthcare wearables compared to their male counterparts (odds ratio [OR] = 1.65, *p* < 0.05). Furthermore, wearable use was found to decline with increasing age. In comparison to CVD patients in the 18–34 age group, those aged 50–64 (OR = 0.32, *p* < 0.05), 65–74 (OR = 0.25, *p* < 0.01), and over 75 (OR = 0.24, *p* < 0.01) were less likely to use healthcare wearables. Regarding race/ethnicity, non-Hispanic Black or African-American patients (OR = 0.17, *p* < 0.01) and Hispanic patients (OR = 0.14, *p* < 0.01) exhibited a lower likelihood of using healthcare wearables in comparison to non-Hispanic White patients.

Significantly, annual household income emerged as a predictor of healthcare wearable use. CVD patients with annual household incomes in the range of 50–75K (OR = 2.04, *p* < 0.01) and above 75K (OR = 2.99, *p* < 0.01) were more likely to use healthcare wearables than those with lower income levels.

In addition to the significant socio-economic demographic predictors mentioned above, it is worth noting that our study did not find any significant association between educational attainment and wearable use among CVD patients. This suggests that education level may not play a substantial role in determining the adoption of healthcare wearables in this population.

Our findings highlight the importance of considering socio-economic factors when examining wearable use among CVD patients. The disparities observed based on gender, age, race/ethnicity, and income level underscore the need for targeted interventions and tailored approaches to ensure equitable access and utilization of healthcare wearables.

In our analysis, we identified two health-related factors that were statistically significant in relation to wearable use among CVD patients. Firstly, CVD patients who perceived their general health to be better demonstrated a higher likelihood of utilizing healthcare wearables (OR = 1.45, *p* < 0.01).

Furthermore, our findings indicate that CVD patients who enjoy exercising exhibit a greater propensity to adopt and use healthcare wearables (OR = 1.76, *p* < 0.01). This suggests that a positive attitude towards exercise may serve as a motivating factor for the adoption and regular usage of wearable devices among CVD patients.

Interestingly, the presence of additional comorbid conditions, such as diabetes or high blood pressure, did not appear to have a significant impact on wearable use. This implies that the presence of these conditions alone may not necessarily motivate CVD patients to engage with healthcare wearables.

In our analysis, we found that CVD patients with higher levels of technological self-efficacy were more likely to utilize wearable devices. Specifically, patients who actively searched the internet for health information (OR = 2.10, *p* < 0.01) and those who made appointments with their healthcare providers electronically (OR = 2.35, *p* < 0.01) demonstrated a higher propensity to use healthcare wearables. These findings highlight the significance of technology literacy and comfort levels with technology in promoting the adoption and utilization of healthcare wearable devices among CVD patients. Patients who possess greater confidence in their ability to navigate and utilize technology are more inclined to incorporate wearable devices into their healthcare routine.

## 4. Discussion 

Despite the growing popularity and sales of smartwatches and activity trackers in the USA, our study highlights that only a small proportion (18.34%) of CVD patients reported using healthcare wearable devices in the prior 12 months. Although 30% of US adults [18] and 25% of Canadian adults [24] have reported using wearable devices, the proportion of CVD patients using wearables seems relatively less. Low or poor use of health wearables or activity trackers among heart patients has been documented by prior studies as well [25]. This indicates a critical need to explore barriers and challenges that hinder wider adoption among this specific patient population. In this study, a significant percentage (41.92%) of individuals with CVD who utilize wearable devices reported using them on a daily basis. These findings, when compared with previous research indicating that 47.3% of adults in the United States and 57% of Canadian adults reported frequent usage of such devices, indicate relatively lower utilization of wearables in the CVD patient groups. In addition, a considerable number (21.56%) did not use it at all. Therefore, understanding the reasons behind these usage patterns can provide insights into optimizing engagement and adherence to wearable technology.

A significant finding from our study is that a majority (83.54%) of wearable users expressed their willingness to share health data from wearables with their healthcare providers. This suggests that CVD patients recognize the potential benefits of sharing wearable data for improving their care and treatment outcomes. Leveraging this willingness can foster more collaborative patient-provider relationships and facilitate the integration of wearable data into healthcare interventions.

Our study notes significant socio-economic and demographic factors that affect healthcare wearable use among CVD patients. The gender disparity observed in the adoption of healthcare wearables among CVD patients is consistent with previous research. Prior studies have also found that women are more likely to adopt and use healthcare wearables, such as fitness trackers [18, 33], and health-related mobile applications [34]. These findings suggest that women perceive significant benefits in using wearables. Such devices provide women with valuable insights into their physical health and enable them to engage in informed decision-making regarding their health practices. Women tend to be more health-conscious and proactive about their well-being, and are typically more interested in tracking their vital signs and fitness levels to maintain a healthy lifestyle [35, 36]. The knowledge gained from healthcare wearables empowers women to make informed choices that promote their overall wellbeing [37].

Our study, in line with previous research, found that older individuals with cardiovascular diseases are less likely to adopt and use healthcare wearables. This is noteworthy because wearables have demonstrated potential benefits for older adults with CVD [38], such as heart rate monitoring, assessment of heart rate variability [39], and tracking cardiorespiratory fitness [40]. One possible explanation for the low adoption rates among older CVD patients is technology anxiety and resistance to change. Studies [21, 41] have indicated that older adults often experience anxiety and apprehension when it comes to using new health technologies. This anxiety can stem from a lack of familiarity with technology, concerns about privacy and data security, or challenges in navigating complex user interfaces or in troubleshooting the device. Wearable devices hold immense potential benefits for older adults who tend to have CVD problems; however, the adoption of wearables within this demographic remains limited [28].

This study revealed notable racial and ethnic disparities in the use of healthcare wearable devices among CVD patients. Specifically, compared to non-Hispanic Whites, both Hispanics and African American CVD patients exhibited a lower likelihood of using healthcare wearables. These findings align with prior research that has documented similar disparities in the adoption of other health-related technologies [42, 43]. CVD risk factors have been found to be higher among African Americans and Hispanics in the USA. Prevalence of hypertension is higher among non-Hispanic African Americans [44] as compared to other groups. Hispanics have the highest prevalence of diabetes, followed by non-Hispanic African Americans and Whites [45]. In terms of obesity, non-Hispanic African Americans have the highest prevalence, followed by Hispanics and Whites [46]. Given such CVD risk factors among African American and Hispanic subgroups, wearables offer immense potential to gather health data and provide appropriate interventions. However, the observed racial and ethnic disparities in healthcare wearable adoption in our study raise important concerns regarding equitable access to and utilization of these technologies among diverse populations. Several possible reasons could be attributed to these disparities. Racial and ethnic minority groups often face socioeconomic challenges, including lower income levels, limited access to healthcare resources, and reduced health literacy [47, 48, 49]. Further, cultural beliefs, preferences, and trust in technology and the healthcare system can influence adoption rates among different racial and ethnic groups. Some communities may have cultural norms or beliefs that shape their attitudes towards technology, including healthcare wearables. In addition, digital literacy levels among certain racial and ethnic groups may hinder individuals from using healthcare wearables. Demographic groups such as African Americans and Hispanics that are more affected by cardiovascular care disparities frequently encounter challenges in digital health literacy and the effective use of eHealth resources [50].

Our analysis revealed that income levels are a significant predictor of wearable device use among CVD patients. This suggests that affordability may be a barrier for individuals with lower income, as wearables and activity trackers may not be financially accessible to them.

A noteworthy finding from our study is that CVD patients who perceive their health as good or better are more likely to use healthcare wearables. It appears that individuals with better overall health have a higher propensity for adopting wearables. Prior research has documented negative association between self-reported health conditions and intentions to use healthcare wearables [51]. Additionally, we did not observe a significant association between the presence of diabetes or high blood pressure and the use of wearables among CVD patients. Furthermore, our findings indicate that CVD patients who have a positive attitude toward fitness and enjoy exercising are more likely to adopt and use wearables. In summary, our study suggests that both generally healthy CVD patients and those with a positive attitude towards fitness are more inclined to be active users of healthcare wearables.

Our study highlights the significant impact of digital literacy on the use and adoption of wearable devices. Similar to prior research that has documented the importance of digital literacy [52, 53, 54], we found that CVD patients who are regular users of digital platforms and tools are more likely to utilize wearable devices. This finding emphasizes the importance of having a certain level of comfort and familiarity with digital tools, as many wearable devices require the use of associated smartphone apps for optimal functionality. Ensuring that individuals have the necessary digital literacy skills is crucial for them to effectively utilize wearables and engage with the accompanying apps. Our study also has implications for makers of healthcare wearables that they need to take into account the disparities in the digital literacy levels of potential users when designing the devices.

Wearable devices have the potential to empower CVD patients and promote active engagement in their own healthcare. Reimbursing these devices acknowledges the value of patient-generated health data and encourages individuals to take an active role in monitoring and managing their conditions. Moreover, wearable devices generate a wealth of health data that can be utilized for research, population health management, and personalized medicine. By reimbursing these devices, policymakers can support the collection of real-world data that can inform evidence-based decision-making, improve clinical guidelines, and facilitate advancements in healthcare technologies.

The low-levels of wearable use among CVD patients and associated factors raise considerable concerns about promoting their use through some policy-level changes and interventions. In recent years, there have been significant changes in national and state-level policies for reimbursement for remote patient monitoring (RPM). RPM typically requires the use of wearables and/or associated mobile applications. In light of COVID-19 pandemic, the Centers for Medicare and Medicaid Services (CMS) expanded reimbursement for RPM services, allowing its use for both acute and chronic conditions. The updated Current Procedure Terminology (CPT) codes allow reimbursement for reviewing RPM data, patient consultations, and training on RPM technologies [55]. Over 36 U.S. states have framed reimbursement policies for RPM, though with some restrictions. Additionally, Medicare has expanded its billable codes for RPM, incorporating remote physiological monitoring and remote therapeutic monitoring as specific forms of RPM [56, 57]. Though smart watches may not be directly reimbursable under Medicare or Medicaid, certain wearable sensors and/or mobile applications that are associated with RPM could qualify for reimbursement. The digital health reimbursement landscape is rapidly evolving, with some private insurers providing coverage for specific wearable devices and digital health solutions. However, more policy changes are needed to spur wearable adoption and use.

### Limitations

It is important to consider several limitations when interpreting the results of this research. Firstly, the study utilized data from a national survey conducted by the National Cancer Institute in the US, which may not have been specifically designed to comprehensively assess wearable device usage. Instead, a subset of data from a larger study was used to gather insights on wearables. We used data from cycles 3 and 4 of the survey and a fruitful extension of this work could incorporate analysis of additional data from future cycles of the survey. Another important limitation of this study pertains to use of proxy measures for some of the variables used in this study. We assessed digital literacy and comfort level with technology using certain measures in-built in the HINTS survey. Digital literacy has been assessed in a more comprehensive way and future survey work could use more established measures [53]. Another limitation is that the data used in the study pertains to US respondents and the results may not be generalizable to other national contexts. Further, wearable use could change over time, and the cross-sectional nature of the study does not capture the changes in patterns of wearable use over time. We were also limited by the set of variables that were captured by HINTS. Data on other pertinent variables such as health literacy or other health complications that HINTS did not gather, were not incorporated in our analysis. Additionally, the reliance on self-reported data introduces the possibility of subjective interpretation by respondents, which could affect the accuracy and reliability of the findings.

## 5. Conclusions

In conclusion, the use of wearable devices among cardiovascular patients holds significant potential for improving healthcare outcomes and patient empowerment in prevention and management of CVD. This research has shed light on various socio-economic and demographic factors, and health and technology related predictors that influence the adoption and utilization of wearable devices in this population. Gender, age, race/ethnicity, and income levels have emerged as significant predictors of wearable use, highlighting the need for targeted interventions to address disparities and promote equitable access to these technologies. Additionally, the findings underscore the importance of digital literacy, positive health perceptions, and enjoyment of exercise in facilitating wearable adoption. As healthcare policy makers and leaders, it is crucial to consider these insights when designing health policies, reimbursement strategies, and educational initiatives to promote the widespread and effective integration of wearable devices into cardiovascular care. By addressing barriers and implementing evidence-based interventions, we can harness the potential of wearable technologies to advance cardiovascular health and improve patient outcomes in a rapidly evolving digital era.

## Data Availability

Data used in this study is comes from the Heath Information National Trends Survey (HINTS) conducted by National Cancer Institute. The data is available at HINTS website (https://hints.cancer.gov/data/default.aspx).
